# The Status of the Workhouse Medical Officer

**Published:** 1912-10-26

**Authors:** 


### THE STATUS OF THE WORKHOUSE MEDICAL OFFICER.

### Dr. C. Thackray Parsons, Medical Superintendent of Fulham Infirmary, on the Opportunity of To-day.

The issue of the Draft Poor-Law Order by the Departmental Committee, which has already excited correspondence and criticism in The Hospital, brings once more the need for reform in the Poor-Law Medical Service to the front, and with a view to having that need explained, and in order that the present position of affairs may be set out authoritatively, our Commissioner asked Dr. Parsons, medical superintendent of the Fulham Poor-Law Infirmary, to give his views on the subject.

"You will remember," Dr. Parsons began, "that the Draft Poor-Law Order refers only to the sick-wards of workhouses, and not to separately administered infirmaries at all. Nevertheless, infirmary medical superintendents are deeply interested in it, not only because many of them are medical officers of workhouses, and all of them are heads of training schools which supply many of the trained nurses employed in workhouses, but also because the Draft Order brings once more into light certain conditions which affect the whole internal Poor-Law Medical Service, and the amendment of which would make it more attractive and more efficient than it is at present."

"What is the present position?"

"At present there are three systems at work. There are the separated infirmaries, in which the medical superintendent is entirely responsible for the administration. Then there are the sick-wards in the workhouse; and, lastly, the sick-wards which are in a separate building in the workhouse grounds. In these last two the workhouse master is, with few exceptions, responsible for the general administration. In the small workhouses, which include one or more sick-wards, there is usually a visiting medical officer, but the workhouse master is supreme. He is also generally responsible in the case of the larger workhouses where there are one or more resident medical officers with or without a visiting staff. Only in the separated infirmaries is the medical superintendent entirely responsible, but that is the goal to aim at, as I shall presently explain.

"The Draft Order is an attempt to codify and simplify the existing scattered Orders and Letters that concern workhouse administration. It has been submitted, I understand, to a number of Poor-Law societies for criticism and suggestions. It is curious, considering the number of medical questions involved, that the Departmental Committee—assuming the names published are correct and complete—does not include any medical man amongst its members."

### Causes of Friction.

"Being a Draft merely the publication may be considered premature?"

"Certainly, but the fact of publication before the final terms have been decided on should help to awaken Poor-Law medical officers to the fact that now or never is the time to present their proposals for reform. Some that they would at once suggest have indeed been embodied in the Draft. Notably the powers of the medical officer and superintendent nurse in the sick-wards are improved. Under the Nursing in Workhouses Order, 1897, the superintendent nurse was subject to the directions of the master and matron as regards the sick-wards on all points except the treatment of the sick. Under the Draft Order she is made responsible for the linen, bedding, and clothing in the sick-wards, for their cleanliness and, subject to the directions of the medical officer, for their heating and ventilation. In addition, the workhouse matron, who is usually untrained in nursing, is to be excluded from the sick-wards."

"This will remove friction?"

"It should certainly do so. Hitherto the workhouse master, who naturally cannot view matters from the point of view of the sick inmate, has often complained of the expense caused by the additional heating which is asked for in the sick-wards, and by what he regards as their extravagant demands for linen. Friction has been caused frequently by the workhouse master having the heating of the sick-wards cut off or reduced, because he thought they were warm enough when he passed through, and medical officers, on protesting, have often been met with such a remark as this: 'What is the use of my heating the wards if you open the windows!' The workhouse masters are fond of comparing the amount of expense incurred in the healthy and in the sick-wards, and do not appreciate how great the difference must necessarily be. A healthy ward may be fully supplied with an amount of linen that means starvation in a sick-ward. The workhouse master's duty is to study economy; he regards the sick-wards as extravagant in the heating, cleaning, and supply of clean things, and their demands and his objections in these matters form the basis of misunderstandings and quarrels. It is, therefore, a great improvement that the superintendent nurse, according to the Draft Order, should be responsible for the warmth, cleanliness, and ventilation of the sick-wards. Given a woman of character, under this regulation much could probably be gained, but in carrying it out there might be some friction. The linen, bedding, and clothing would be given to the superintendent nurse, but she may have to fight for her proper amount of supplies, because the workhouse master still remains her direct superior. The Draft Order, indeed, does not go so far as the Local Government Board has already gone in some Unions, where by special orders both the workhouse master and matron have been excluded from the sick-wards. Again, the Draft Order says the superintendent nurse must report to the master any misconduct in the wards, while there is nothing that says she must report it to the medical officer. That places the medical officer in an impossible position. The medical officer must know directly of any misconduct that occurs in the sick-wards, and he must have powers to suspend the person responsible if necessary."

### The Essential Reforms.

"You would suggest?"

"The workhouse master should be relieved of all administrative responsibility in the sick-wards, no matter where they may be situated. The medical officer should be responsible to the House Committee for their entire administration. He should be in the sick-wards in the workhouse what the medical superintendent is in the separately administered infirmary. Then, again, the sick-wards must be confined to sick inmates, as is not always the case in the overcrowded workhouses. Were this rigidly enforced there would be no reason for the workhouse master to intervene. Overcrowding is found in the workhouses of the large cities and even in some of the separate infirmaries in London. The Draft Order should make it clear that the sick-wards should be reserved for the sick only, and that no inmate should be warded in them except by the directions of the medical officer."

"The present is a favourable opportunity for presenting the medical officer's views?"

"Yes, for if the Draft Order be issued it will not be amended for a considerable time. The difficulty is the want of cohesion among medical officers. There is no association of workhouse medical officers as such. The Poor-Law Medical Officers' Association contains so many district medical officers that the workhouse medical officers' position is apt to be overlooked. The Association is, however, taking great interest in this matter, and is taking steps to bring its views before the Departmental Committee. Then there is another body, the Medical Superintendents' Association, which is confined to resident superintendents of separated infirmaries. Many of its members are medical officers of adjoining workhouses, and the Association hopes that it will be allowed to submit its criticisms on the Draft Order to the Committee. Whether the Draft Order will be amended remains to be seen, and whether, if so, it will get beyond the Local Government Board is another matter."

### The Question of Salary.

"Apart from the Order, what changes do you think essential?"

"The present salary of the workhouse medical officer is inadequate, as indeed was reported by the recent Royal Commission. The worst feature of his present remuneration is that in many cases it includes payment of the medicines he supplies. This means in practice that a large part of his salary is spent in providing drugs for the sick inmates. The Local Government Board knows this and is opposed to the system; it has tried to induce the Guardians to supply the necessary drugs, but in many cases they have refused. A telling illustration of the evils caused by insisting on the medical officer purchasing them out of his salary was given by Dr. Fuller in his evidence before the Royal Commission. He relates how on visiting a workhouse and seeing a syphilitic patient, he inquired of the medical officer whether he was treating him with drugs. 'I cannot afford it,' was the reply. 'I think,' added Dr. Fuller, 'that is a very good example of cases that frequently come under my notice.' * The medical officer at present cannot afford to buy up-to-date and expensive drugs, and his inability to do so may cost the life of an inmate in the workhouse.† Again, some witnesses alleged that owing to the small salary the medical officer gave insufficient attendance to the sick inmates. The evidence placed before the Commission leaves no doubt that this has happened in many places. On the other hand, evidence was given showing that medical officers in many cases devoted themselves to their duties in a most self-sacrificing manner, and employed a large proportion of their meagre salaries in the purchase of the necessary drugs for the treatment of the sick. The proper course is for the Guardians to provide all the drugs and medical or surgical appliances required. Now the Draft Order increases the work of the medical officer considerably, and if this work is to be carried out his salary must be increased, or he must be provided with assistance. The medical officer is to keep records of the physical condition of the inmates and bed-cards describing the progress of the sick. This is the practice in every separated infirmary, and is often compulsory; it is never compulsory in the workhouse, where no medical records are kept except those in the Medical Relief Register, where the name, diagnosis, and length of stay are jotted down."

"The Draft Order refers to amount of attendance?"

"Yes; it makes a proposal which, I think, will defeat its own end. It lays down the number of times when the medical officer must examine children. It says that every infant—that is, every child under eighteen months—must be examined once in two weeks; that every child—that is, one between the ages of three and sixteen—must be examined once a month. In short, it prescribes an amount of medical attendance far in excess of what any private child receives. If the child is healthy, such an amount of medical superintendence is quite unnecessary, while if the child is unhealthy it is absurdly inadequate. Therefore it would be much better to leave the medical officer free to examine the children when he thinks necessary, and to make him responsible for the administration of the nurseries. No doubt workhouse nurseries would benefit by more medical supervision. To get it, place them under the medical officer, and his own pride will lead him to do his best."

### The Conditions of the Service.

"Then, as to the present conditions of the Poor-Law Indoor Medical Service?"

"At present the conditions are not such as to attract the best man, and you will only get him by making them sufficiently attractive. The Outdoor Service is often attractive to private practitioners; the Indoor is not nearly attractive enough; indeed, the only attraction offered by its junior posts is experience, and unless the workhouse is well administered and well supplied with appliances for investigating disease and for treating it, the value of the experience is greatly reduced. The dearth of candidates for the Service is, therefore, explained by the small salary and the small prospects. The chances of a medical superintendentship are small, and after he has been in the Service two or three years the prudent young medical officer begins to ask himself what the future holds for him, and finds no adequate reply. The Poor-Law Service in these respects compares very unfavourably with the Lunacy Service, the Public Health Service, or the Fever Service under the Metropolitan Asylums Board. For the post of workhouse resident medical officer in the smaller Unions it is really a wonder than anyone applies. For general practice the experience offered by a Poor-Law infirmary is very good, and much better than that afforded by the hospitals. Minor cases, such as will be met in general practice, chronics, and nervous cases, which are turned out of the hospitals, are all to be seen by the infirmary medical officer. There is acute work as well, and the junior officer in the Poor-Law Service has to act practically on his own responsibility, far more so than in the hospital resident posts. With an infirmary, for instance, of 500 to 800 beds it is physically impossible for the medical superintendent to know every case. The treatment of many of the cases has to be left in the hands of the medical officer. This trains his junior officers in self-reliance. And he also has many opportunities of performing operations, such as for strangulated hernia and acute appendicitis—operations which he may at any time have to face on his own responsibility in private practice. Still there is sometimes no proper provision for operations, no operating room, and no apparatus for electrical treatment or massage, unless by accident one of the sisters has had some training in this respect. Under these circumstances how can you expect to attract the best men? Many Guardians do not appreciate the fact that it is cheaper to spend money in perfecting the treatment so that patients may be cured and then got rid of, than to keep them as chronics at continual expense."

---

* Evidence before the Commission. Questions 10,610-1.
† *Ibid.* Q. 4,943.

"The workhouse master influences the Board?"

"In the case of the workhouse sick-wards as distinct from the separate infirmaries—Yes. He is always present at committee meetings while the medical officer is not; he generally has the ear of the Guardians, and can always suggest that the medical officer's proposals will increase the expenditure. The crux of the whole matter is really the individuality of the medical officer. The Service is not attractive enough for the good men, and some medical officers are not firm enough. They must be capable of holding their own, of knowing their work thoroughly, of appreciating what is required, and of insisting in season and out of season until they get it. A capable man, who remembers the lesson of the importunate widow, and does not allow questions to drop after they have been raised, can generally get his way in the end. The Service, therefore, must be made more attractive. The improvements hitherto gained have been won by men who have fought on till they got the improvements they wanted. But there must be hopes in prospect to encourage such men. The better posts are few, and are awarded by open competition, by Boards who know nothing of a man's personal record, and are often swayed by purely personal considerations. Outsiders, therefore, are sometimes appointed with none of the claims of the hard-working and conscientious officer."

Enlarge the Areas.

"What reform would obviate this?"

"The administrative areas should be enlarged. There is now no co-operation between the thirty-one Boards of Guardians of London. To secure efficiency in appointments, and to give possibilities of promotion, combined with a knowledge in the authority of the personal qualities of every candidate, these Unions should be included under one administration with local committees in each case. The neighbouring Urban Unions, such as West Ham, Richmond, Brentford, and Croydon, might be included. This would give a good Service, with those chances of promotion that are essential to efficiency, for every medical officer's work would be known to the central office."

"Would the same centralisation be carried out in the provinces?"

"I do not see why each large town should not be organised on the same principle, but the difficulty would be in the rural districts. These difficulties, however, should not prove insuperable. One central infirmary might deal with the sick, though the question of distance would have to be considered. Such a central infirmary would have to be sufficiently accessible for the removal of sick patients and for visits to be paid to them by relatives and friends. In any case, much would be accomplished if London and the larger towns made a start on these lines. At present the Poor-Law Indoor Medical Service is not attractive enough to secure the best men, and it will not do so until the administration of the sick-wards is separated from that of the general workhouse and is given into the hands of the medical officer, and the Poor-Law Medical Service is organised in large areas so that the provision of properly constructed and well-equipped infirmaries becomes economically possible and the medical officer feels that the Service offers him adequate remuneration and a fair prospect of promotion."

At a meeting of the Manchester Branch of the Incorporated Association of Hospital Officers on Thursday, last week, Mr. E. Lionel Blake was unanimously elected president for the ensuing year. Mr. Blake is superintendent-secretary of the Royal Infirmary, Oldham.

